# Identification of Phenolic Compounds by LC-MS/MS and Evaluation of Bioactive Properties of Two Edible Halophytes: *Limonium effusum* and *L. sinuatum*

**DOI:** 10.3390/molecules26134040

**Published:** 2021-07-01

**Authors:** Ipek Baysal, Melike Ekizoglu, Abdulselam Ertas, Burak Temiz, Hale Gamze Agalar, Samiye Yabanoglu-Ciftci, Hamdi Temel, Gulberk Ucar, Fatma Pinar Turkmenoglu

**Affiliations:** 1 Vocational School of Health Services, Hacettepe University, 06100 Ankara, Turkey; ipekbaysal@hacettepe.edu.tr; 2 Department of Pharmaceutical Microbiology, Faculty of Pharmacy, Hacettepe University, 06100 Ankara, Turkey; melike@hacettepe.edu.tr; 3 Department of Pharmacognosy, Faculty of Pharmacy, Dicle University, 21280 Diyarbakir, Turkey; abdulselam.ertas@dicle.edu.tr; 4 Department of Pharmacognosy, Faculty of Pharmacy, Anadolu University, 26470 Eskişehir, Turkey; burak_temiz@anadolu.edu.tr (B.T.); hgduymus@anadolu.edu.tr (H.G.A.); 5 Department of Biochemistry, Faculty of Pharmacy, Hacettepe University, 06100 Ankara, Turkey; samiye@hacettepe.edu.tr (S.Y.-C.); gulberk@hacettepe.edu.tr (G.U.); 6 Department of Pharmaceutical Chemistry, Faculty of Pharmacy, Dicle University, 21280 Diyarbakir, Turkey; htemel@dicle.edu.tr; 7 Department of Pharmaceutical Botany, Faculty of Pharmacy, Anadolu University, 26470 Eskişehir, Turkey

**Keywords:** *Limonium effusum*, *Limonium sinuatum*, Plumbaginaceae, phenolics, LC-MS/MS, antioxidant, antimicrobial, anticholinesterase, antityrosinase, pancreatic lipase, enzyme inhibition

## Abstract

This work aimed to evaluate the phenolic content and *in vitro* antioxidant, antimicrobial and enzyme inhibitory activities of the methanol extracts and their fractions of two edible halophytic *Limonium* species, *L. effusum* (LE) and *L. sinuatum* (LS). The total phenolic content resulted about two-fold higher in the ethyl acetate fraction of LE (522.82 ± 5.67 mg GAE/g extract) than in that of LS (274.87 ± 1.87 mg GAE/g extract). LC-MS/MS analysis indicated that tannic acid was the most abundant phenolic acid in both species (71,439.56 ± 3643.3 µg/g extract in LE and 105,453.5 ± 5328.1 µg/g extract in LS), whereas hyperoside was the most abundant flavonoid (14,006.90 ± 686.1 µg/g extract in LE and 1708.51 ± 83.6 µg/g extract in LS). The antioxidant capacity was evaluated by DPPH and TAC assays, and the stronger antioxidant activity in ethyl acetate fractions was highlighted. Both species were more active against Gram-positive bacteria than Gram negatives and showed considerable growth inhibitions against tested fungi. Interestingly, selective acetylcholinesterase (AChE) activity was observed with LE and LS. Particularly, the water fraction of LS strongly inhibited AChE (IC_50_ = 0.199 ± 0.009 µg/mL). The ethyl acetate fractions of LE and LS, as well as the *n*-hexane fraction of LE, exhibited significant antityrosinase activity (IC_50_ = 245.56 ± 3.6, 295.18 ± 10.57 and 148.27 ± 3.33 µg/mL, respectively). The ethyl acetate fraction and methanol extract of LS also significantly inhibited pancreatic lipase (IC_50_ = 83.76 ± 4.19 and 162.2 ± 7.29 µg/mL, respectively). Taken together, these findings warrant further investigations to assess the potential of LE and LS as a bioactive source that can be exploited in pharmaceutical, cosmetics and food industries.

## 1. Introduction

Halophyte species, which can be found in environments such as sand hills, deserts, cliffed coasts, saline lakes, coastal and inland salt marshes, are naturally adapted plants to high levels of soil salinity [1]. It is known that soil salinity and drought, which are the major abiotic stress in plants, cause overproduction and the accumulation of reactive oxygen species (ROS), which are toxic and highly reactive. ROS can interact with vital molecules and damage nucleic acids, proteins, carbohydrates, lipids, etc., which eventually result in oxidative stress. In addition, ROS may have a key role as signaling molecules that modulate a wide range of diverse plant processes under salt stress.

Apart from being able to regulate the ion and water movements, halophytes, due to their robust enzymatic and non-enzymatic antioxidant systems, are also known for their ability to quench ROS [2]. On the other hand, it is a fact that the synthesis and accumulation of polyphenols in halophytic plants are usually stimulated in response to both biotic and abiotic stresses [3]. Nowadays, phenolic compounds are at the center of attention, as they play a crucial role in preventing oxidation processes and have highly valued functions in improving health and preventing disturbances ranging from cancer, inflammation, high blood pressure, neurodegenerative disorders, and obesity to skin disorders and acute oxidative damage, etc. [4,5]. Therefore, besides halophytes, phenolic extracts hold great importance, due to their variety of applications in fields such as the food industry, cosmetics, pharmaceuticals and medicine [2,5,6].

The genus *Limonium* Mill. (sea lavender), which belongs to Plumbaginaceae, consists of approximately 350–600 halophytic species of usually perennial herbs and dwarf shrubs adapted to saline soils. The genus has a cosmopolitan distribution; however, the center of diversity of the *Limonium* is the Mediterranean region [7]. Traditional folk medicine studies indicate that some *Limonium* species have been used for various medical purposes all over the world. For example, in China, *L. sinense* (Girard) Kuntze is used for curing fever, hemorrhaging, hepatitis, bronchitis, diarrhea, etc. [8], while *L. michelsonii* Lincz., which is native to the Almaty region of Kazakhstan, is used for the treatment of poor blood circulation [9]. On the other hand, in Argentina, Uruguay and Brazil, *L. brasiliense* (Boiss) Kuntze (local name: baicuru, guaicuru) have been used for menstrual disorders and cramps as an antiseptic for genitourinary infections [10], while *L. wrightii* O. Kunthe., which grows in the seashore of the Okinawa Islands (local name: ukonisomatsu), is used for the treatment of fever and arthritis [11].

A literature survey revealed that the *Limonium* species is rich in phenolics and terpenoids. Polysaccharides, aliphatic compounds, amino acids and minerals were also reported [8,9]. Bioactivity studies have shown that various *Limonium* species possess free radical scavenging, antioxidant, antimicrobial, antiviral, anti-inflammatory, anticancer, immunomodulatory, hepatoprotective, anti-melanogenic, anti-obesity and enzyme inhibitor activities, and so on. [8,9,10,11,12,13]. In addition to the therapeutic importance of the genus *Limonium*, some species have ornamental value because of attractive flowers, some are consumed as foods and some are used as antioxidants in cosmetic products [14].

The genus *Limonium* is represented by 27 halophytic taxa in Turkey [7]; among them, two species, *L. effusum* (Boiss.) O. Kuntze (Turkish name; kaya marulu) and *L. sinuatum* (L.) Miller, (Common name; statis, sea lavender, Turkish name; deniz out, deniz marulu, herdemtaze, limonotu, sahilkaranfili) are edible. Both species grow wildly in seashores, sand dunes or rocky coasts. *L. effusum*, which is endemic, also grows in salty hinterlands of the Aegean and Mediterranean regions of Turkey. Aerial parts of both species, sometimes only leaves or young shoots, are eaten fresh, added to salad or pastry, consumed as soup, meal and roasted in Turkish cuisine as a part of the Mediterranean diet [15,16,17]. Considering that Turkey has the longest Mediterranean coastline, the popular Mediterranean diet, for which wild edibles constitute a major element, is one of the essential components of Turkish cuisine. *L. sinuatum* is also consumed raw in salad in various European countries, such as Greece and Cyprus, and used as a fresh or stewed vegetable in Spain [18,19]. To the best of our knowledge, data on the *Limonium* species growing in Turkey are still limited. Having in mind the possible high antioxidant capacity of halophytes, which are a promising source of drugs, cosmeceuticals and nutraceuticals for the pharmaceutical, cosmetic and food industries, in this study, the *in vitro* antioxidant, antimicrobial, acetyl (AChE) and butyrylcholinesterase (BChE), tyrosinase and pancreatic lipase inhibitory activities of methanol extracts and their *n*-hexane, dichloromethane, ethyl acetate and water fractions, prepared from aerial parts of *L. effusum* and *L. sinuatum*, were evaluated. In addition, the total phenolic content of crude extracts and their fractions were determined, and the phenolic compounds of total phenolic rich ethyl acetate fractions were qualified and quantified by a LC-MS/MS analysis.

## 2. Results and Discussion

### 2.1. Determination of Total Phenolic Content

The total phenolic content (TPC) of extracts was determined by the Folin–Ciocalteu colorimetric method, which measures the level of total phenolics based on oxidation-reduction mechanisms [20]. Table 1 shows the TPC as well as antioxidant activity of *L. effusum* and *L. sinuatum.* The content of total phenolics is expressed as gallic acid equivalents (GAE, mg gallic acid/g extract). In the present study, TPC varied from 75.98 ± 0.88 to 522.82 ± 5.67 mg GAE/g extract of the samples and, among them, the highest TPCs were found in ethyl acetate fractions. Significantly, TPC resulted as about two-fold higher in the ethyl acetate fraction of the methanolic extract of *L. effusum* (522.82 ± 5.67 mg GAE/g extract), than that of *L. sinuatum* (274.87 ± 1.87 mg GAE/g extract).

According to the literature survey, the present study provides the first report on TPC of *L. effusum.* In a recent study, TPC values for methanol extracts prepared from aerial parts and roots of *L. sinuatum* were found to be 145.96 ± 0.36 and 93.60 ± 1.16 mg GAE/g extract, respectively [21]. In this study, the TPC of *L. iconicum, L. globuliferum, L. lilacinum*, *L. gmelinii*, *L. bellidifolium* were also reported (aerial parts: ranging from 43.63 ± 0.19 to 116.51 ± 1.40 mg GAE/g extract) (roots: ranging from 47.48 ± 1.10 to 238.18 ± 2.43 mg GAE/g extract). In another study, Li et al. [22] reported that the TPC values of the water soluble and fat soluble fractions of the tetrahydrofuran extract of *L. sinuatum* and the combination of these fractions were 22.84 ± 0.83, 11.34 ± 0.34 and 37.17 ± 1.17 mg GAE/g extract, respectively, which are much lower than the values obtained in this study. However, in this screening study [22], *L. sinuatum* was reported to be one of the most prosperous species in terms of phenolic content among 51 plant species from China. In addition, the TPC of *n*-hexane, acetone:water (8:2), methanol:water (8:2) and water extracts of *L. delicatulum* shoots were reported as ranging from 0.19 ± 0.03 to 92.9 ± 1.45 mg GAE/g extract when plants are at the flowering period, and from 0.13 ± 0.05 to 44.13 ± 3.43 mg GAE/g extract at the vegetative stage [23]. All these previous data are in agreement with the high phenolic content of the *Limonium* species.

### 2.2. Identification of Phenolic Compounds by LC-MS/MS

Twenty-four phenolic compounds, including phenolic acids, phenolic aldehyde, flavonoids, flavonoid glycosides, and coumarin, as well as three non-phenolic organic acids, which are widespread in edible plants, were analyzed in phenolic-rich ethyl acetate fractions of methanol extracts of *L. effusum* and *L. sinuatum.* The analytical method was previously described by our research group [24,25].

**Figure 1:**
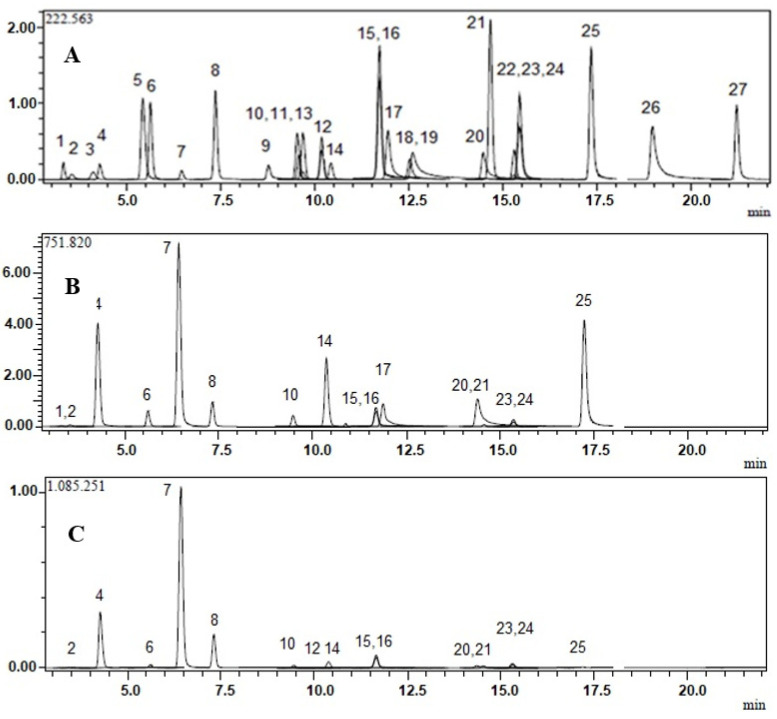
LC-MS/MS chromatograms of (**A**) Calibration 2 level of standard mix. 1: Quinic acid. 2: Malic acid. 3: *trans*-Aconitic acid. 4: Gallic acid. 5: Chlorogenic acid. 6: Protocatechuic acid. 7: Tannic acid. 8: *trans*-Caffeic acid. 9: Vanillin. 10: *p*-Coumaric acid. 11: Rosmarinic acid. 12: Rutin. 13: Hesperidin. 14: Hyperoside. 15: 4-OH Benzoic acid. 16: Salicylic acid. 17: Myricetin. 18: Fisetin. 19: Coumarin. 20: Quercetin. 21: Naringenin. 22: Hesperetin. 23: Luteolin. 24: Kaempferol. 25: Apigenin. 26: Rhamnetin. 27: Chrysin. (**B**,**C**): Ethyl acetate fractions of methanol extracts of *L. effusum* and *L. sinuatum,* respectively.

According to the LC-MS/MS results of the current study, the ethyl acetate fractions of *L. effusum* and *L. sinuatum* had very rich phenolic content because of their high levels of tannic acid (71,439.56 ± 3643.3 and 105,453.5 ± 5328.1 μg/g extract, respectively) and hyperoside (14,006.90 ± 686.1 and 1708.51 ± 83.6 μg/g extract, respectively) content (Figure 1 and Table 2). In terms of the flavonoids, high amounts of myricetin (1646.93 ± 97.1 μg/g extract), quercetin (975.24 ± 69.2 μg/g extract) and apigenin (751.20 ± 39.8 μg/g extract) in *L. effusum* drew attention. Contrary to *L. effusum*, in the ethyl acetate fraction of the methanol extract of *L. sinuatum*, myricetin was not detected. In addition, the amount of quercetin was ten times lower (94.23 ± 6.7 μg/g extract) and apigenin was one hundred times lower (7.49 ± 0.4 μg/g extract) than that of *L. effusum*. In terms of non-phenolic compounds, *L. effusum* and *L. sinuatum* contain high amounts of malic acid (1007.66 ± 53.3 and 410.69 ± 21.7 μg/g extract, respectively). A significant amount of quinic acid (636.08 ± 30.5 μg/g extract) was only detected in *L. effusum* (Figure 1B,C and Table 2).

In terms of the phenolic profile of the *Limonium* species, few data have been reported in the literature. In a previous study regarding *L. sinuatum*, high amounts of catechin (1108.53 mg/100 g), homogenistic acid (837.91 mg/100 g) and epicatechin (602.66 mg/100 g) were reported [22]. In another study, a HPLC-ESI-MS analysis indicated that the most abundant compounds in methanol extracts of *L. delicatulum* and *L. quesadense* leaves were myricetin glycosides (4.40 ± 0.01 mg/g extract) and the mono and dimer of gallo(epi)catechin-*O*-gallate (26 ± 1 and 10.0 ± 0.7 mg/g extract, respectively), respectively [26]. In addition to these studies, few qualitative analyses have been reported; for instance, *p*-coumaric acid, 1,2-*p*-hydroxybenzoic acid, chlorogenic acid, gallic acid, 4,3,5-dimethoxyhydrobenzoic acid and rutin were identified by reverse-phase HPLC in *L. delicatulum* shoot extracts. *p*-Coumaric acid was reported to be the major compound, followed by chlorogenic acid [23]. High concentrations of catechins (epigallocatechin gallate and catechin-3-gallate), chalcons (phlorizin and phloretin), quercetin and myricetin glycosides were reported in *L. contortirameum* and *L. virgatum* [27]. When compared to the literature, data presented in this study are the first report that indicate such high amounts of tannic acid and hyperoside in the *Limonium* species. Within this context, it could be said that both *L. effusum* and *L. sinuatum* are regarded as new sources for tannic acid and hyperoside (Table 2).

### 2.3. Antioxidant Activity

The antioxidant properties of phenolic compounds are well known; DPPH, a relatively stable organic radical, has been extensively used in the determination of the antioxidant capacity of medicinal plant extracts. In the present study, free radical scavenging activity of the extracts was evaluated by the DPPH method [28] compared to that of a synthetic antioxidant, tert-butylhydroxytoluene (BHT); the results are given in Table 1. The IC_50_ value ranged from 13.92 ± 0.06 µg/mL to 227.97 ± 15.83 µg/mL for *L. effusum*, and from 5.27 ± 0.002 µg/mL to 180.50 ± 6.89 µg/mL for *L. sinuatum*. When compared to BHT, the prominent radical scavenging activities, which were lower than BHT, were observed in methanol extracts and the phenolic-rich ethyl acetate fractions of both *L. effusum* and *L. sinuatum* with IC_50_ = 28.72 ± 0.79 µg/mL, IC_50_ = 30.79 ± 0.7 µg/mL, IC_50_ = 30.15 ± 0.82 µg/mL and IC_50_ = 5.27 ± 0.002 µg/mL, respectively. In addition, interestingly, the *n*-hexane fraction of *L. effusum* showed prominent DPPH radical scavenging activity with IC_50_ = 13.92 ± 0.06 µg/mL. It is known that, besides phenolic compounds, some lipid soluble plant metabolites, such as carotenoids and terpenoids, possess strong radical scavenging activity [29]. In this context, the high free radical scavenging activity observed in *n*-hexane fraction of *L. effusum* might probably be due to its lipid-soluble antioxidant metabolite content.

The total antioxidant capacity (TAC) assay is a copper-reducing antioxidant assay based on the reduction of copper (II) to copper (I) by antioxidants, and provides determination of the antioxidant potential of complex samples, including plant extracts. TAC was also reported to be useful for a wide variety of phenolics, such as phenolic acids, flavonoids, coumarins, anthocyanins, carotenoids, etc. [24,30]. The TAC of *Limonium* extracts and fractions at 100 µg/mL concentration are shown in Table 1. According to our results, the highest TACs were observed in the ethyl acetate fractions of both *Limonium* species (*L. effusum*, 0.453 ± 0.007 UAE, and 990.75 ± 14.86 CRE); *L. sinuatum,* 0.836 ± 0.016 UAE and 1829.47 ± 35.27 CRE), which have the highest total phenolic content. Furthermore, the TAC of the ethyl acetate fraction of *L. sinuatum* was found to be higher than BHT (0.540 ± 0.022 UAE and 1182.99 ± 48.37CRE).

A few studies have been reported on the antioxidant potential of the genus *Limonium*. For instance, very recently, potent DPPH scavenging activities of six *Limonium* species ranging from 90.10 to 507.94 mg trolox equivalent/g were reported [21]. In another study, the strong DPPH radical scavenging activity of the water extract of *L. wrightii* (IC_50_ = 500 µg/mL) was reported. In this study, gallic acid was indicated as the active component of *L. wrightii* with a strong free radical scavenging action (IC_50_ = 2.63 µg/mL) [11]. According to Medini et al. [23], 80% acetone (IC_50_ = 2 ± 0.68 µg/mL), 80% methanol (IC_50_ = 5.25 ± 0.62 µg/mL) and 95% ethanol (IC_50_ = 4.3 ± 0.14 µg/mL) extracts of *L. delicatulum* had more potent activity toward DPPH radical than that of BHT (IC_50_ = 11.5 ± 0.2 µg/mL). In addition, hexane extract (IC_50_ > 1000 µg/mL) had the lowest antiradical activity, while the most polar water extract (29 ± IC_50_ = 5.73 µg/mL) had moderate activity. According to these previous data, the presented study reports the significant radical scavenging ability of the lipophilic *n*-hexane fraction of the *Limonium* species for the first time.

Phenolics constitute one of the main bioactive compound groups in plants related to antioxidant activity. In this study, high antioxidant activities were observed in ethyl acetate fractions of both *L. effusum* and *L. sinuatum*, which exhibited the highest TPCs among the tested samples (Table 1). In terms of correlation between the antioxidant activity and TPC, the presented results are in agreement with the literature.

### 2.4. Antimicrobial Activity

Methanol extracts and their fractions of *L. effusum* and *L. sinuatum* were screened for their *in vitro* antibacterial activity against six selected bacteria, *Staphylococcus aureus* ATCC 29213, methicillin resistant *S. aureus* (MRSA) ATCC 43300, *Enterococcus faecalis* ATCC29212, *Escherichia coli* ATCC 25922, *Pseudomonas aeruginosa* ATCC 15442 and *Staphylococcus epidermidis* ATCC 35984, and for their *in vitro* antifungal activity against three *Candida* species, *C. albicans* ATCC 90028, *C. krusei* ATCC 6258 and *C. parapsilosis* ATCC 90018. The Minimum Inhibitory Concentration (MIC, µg/mL) values are given in Table 3. According to our results, methanol extracts and their fractions of both *Limonium* species are more active against Gram-positive bacteria than Gram negatives. *E. faecalis* was the most susceptible bacteria among those tested at the tested concentrations. *n*-Hexane and ethyl acetate fractions of *L. sinuatum* were the most effective against Gram-positive bacteria, with MICs ranging from 16 to 32 µg/mL and 32 to 128 µg/mL, respectively. Lower inhibitory effects were showed by basic methanol extracts; however, the methanol extract of *L. sinuatum* exhibited a remarkable inhibitory effect against *E. faecalis* with MIC 64 µg/mL. *n*-Hexane and ethyl acetate fractions of *L. effusum* were found to be active against *S. epidermidis* (MICs 128 µg/mL) and *E. faecalis* (MICs 64 µg/mL and 128 µg/mL, respectively). Water and dichloromethane fractions of both Limonium species exhibited the lowest antimicrobial activity for almost all bacteria.

Antifungal activity studies of *L. effusum* and *L. sinuatum* showed considerable growth inhibitions against *C. albicans* with MICs ranging from 128 to 256 µg/mL and 64 to 256 µg/mL, respectively, and *C. krusei* with MICs 128 µg/mL and 64 to 128 µg/mL, respectively. *C. parapsilosis* was the most susceptible yeast among those tested at the tested concentrations. *L. effusum* and *L. sinuatum* possessed antifungal activity against *C. parapsilosis* with MICs ranging from 64 to 128 µg/mL and 32 to 64 µg/mL, respectively. The ethyl acetate fraction of *L. sinuatum* was the most effective among the tested samples against three Candida species with MICs ranging from 32 to 64 µg/mL.

Antibiotic resistance is one of the most challenging clinical as well as global health problems; therefore, recently, there has been extreme interest in searching for novel antimicrobial drugs, especially from natural sources. However, in the literature, there are few studies about the antimicrobial activity of *Limonium* species. Avaz et al. [31] reported the antimicrobial properties of the root extracts of *L. globuliferum, L. effusum*, and *L. lilacinum* by the disc diffusion method. According to this study, the methanol extract of *L. lilacinum* exhibited the most potent antibacterial activity among the tested extracts. Among the 9 bacteria used, *E. coli* was not found to be sensitive to extracts. For antifungal activity assay, 12 fungi isolated from walnut and hazelnut were used and the *L. globuliferum* water extract was found to be the most potent extract. In another study [32], it was reported that the methanol extract obtained from *L. socotranum* leaves exhibited higher antibacterial activity against *Micrococcus luteus* (MIC 15.6 µg/mL), *S. aureus* (MIC 125 µg/mL) and *P. aeruginosa* (MIC 125 µg/mL) than the methanolic stem extract. The petroleum ether extract displayed stronger antifungal activity (with MIC of 125 µg/mL) than dichlorometane and methanol extracts. A screening study [33] also showed that aerial parts of *L. morisianum* exhibited potent inhibitions against *S. aureus* (IC_50_ = 9.2 µg/mL) and *S. epidermidis* (IC_50_ = 3.9 µg/mL) among thirty-six plant species tested, which indicated the positive correlation between the antibacterial activities and total phenolic content. Another screening study performed on eight extremorphile plants from Tunisia highlighted the antibacterial activity of the stem and leaves of *L. virgatum* with a broad spectrum (MICs range from 312 to 625 µg/mL). The methylene fraction of *L. virgatum* extracts also exhibited high antibacterial activity, with a selective action against some Gram-positive bacteria, particularly against some *Staphylococcus* and *Streptococcus* strains. This activity could be due to the phenolic amide content of the plant [34].

### 2.5. Acetylcholinesterase and Butyrylcholinesterase Inhibitory Activity

Cholinesterase inhibition is one of the primary approaches to treat neurodegenerative disorders, such as Alzheimer’s disease (AD), which is the leading cause of dementia in people above 60. Epidemiological data indicate that about 34 million people are affected worldwide. In AD patients, levels of the neurotransmitter acetylcholine (ACh) are lower than in healthy people, as it is hydrolyzed by acetylcholinesterase (AChE) as well as butyrylcholinesterase (BChE). The inhibition of these enzymes can cause an increase in ACh levels in the synaptic cleft that improves cognitive functions. Cholinesterase inhibitors, such as tacrine, donepezil, rivastigmin, galanthamine, are used to treat the mild type of AD. However, their side effects limit their use [35]. Therefore, recently, the inhibitory effects of plant extracts, which are a diverse and influential source of active compounds, have attracted great interest among researchers [36].

In the present study, *L. effusum* and *L. sinuatum* extracts and their fractions exhibited selective inhibition against AChE in a dose-dependent manner (results not shown) (Table 4). The lowest IC_50_ value for AChE was with the water extract of *L. sinuatum* (0.199 ± 0.009 µg/mL). BChE was also inhibited by *L. effusum* and *L. sinuatum*, except for the water fraction of *L. effusum.* The highest inhibitions against BChE were recorded with *n*-hexane fractions of both *L. effusum* and *L. sinuatum* species (IC_50_ = 224.03 ± 25.78 and 308.72 ± 9.65 µg/mL, respectively).

The methanol extract of *L. effusum*, and water and methanol extracts of *L. sinuatum* exhibited the best selectivity index toward AChE, which are even better than donepezil.

Few studies were performed on the anticholinesterase activity of the *Limonium* species. Similar to our findings, the methanolic and water extracts of *L. delicatulum* and *L. quesadense* leaves exhibited potent activity against AChE and BChE, ranging from 0.86 mg ± 0.01 galantamine equivalent/g to 4.8 ± 0.7 galantamine equivalent/g, except for the water extract of *L. delicatulum,* which was inactive against BChE [26]. In a previous report, infusions and decoctions prepared from aerial parts of *L. algarvense* exhibited moderate activity on AChE (IC_50_ = 220 ± 0.01 and 390 ± 0.02 µg/mL, respectively) and BChE (IC_50_ = 840 ± 0.04 and 960 ± 0.01 µg/mL, respectively) [37]. Mazouz et al. [38] indicated that methanol and methanol:water extracts of *L. spatulatum* significantly inhibited both AChE (IC_50_ = 31.14 ± 0.44 and 3.28 ± 0.16 µg/mL, respectively) and BChE (IC_50_ = 36.65 ± 0.34 and 26.64 ± 0.96 µg/mL, respectively); however, the chloroform extract of the plant was not active. In a recent study, Senizza et al. [21] reported that the *L. sinuatum* root extract was the most active sample tested against AChE and BChE (5.11 mg galantamine equivalent/g and 10.75 mg galantamine equavalent/g, respectively) among the methanol extracts obtained from the aerial parts and roots of six *Limonium* species. In the same study, the anticholinestrase activity of the aerial parts of *L. sinuatum* was reported to be 4.87 ± 0.14 mg galantamine equivalent/g (AChE) and 4.72 ± 0.98 mg galantamine equivalent/g (BChE).

Natural compounds from different chemical classes, such as alkaloids, coumarins, flavonoids, phenolic acids, terpenes, and stilbenes, were reported to have anticholinesterase activity [36]. On the other hand, besides the individual anticholinesterase activity of some phenolic compounds, such as phenolic, gallic, salicylic, and vanillic acids and flavonoids, such as myricetin, apigenin, and quercetin, which are known, it was also reported that the combination of these different groups of compounds might increase or decrease anticholinesterase activity [38,39]. As a result, different groups of compounds or the synergistic effect of flavonoids and phenolic acids or other compounds may be attributed to the anticholinesterase activity observed in this study.

### 2.6. Tyrosinase Inhibitory Activity

Melanogenesis is responsible in humans for the color of the skin, hair and eyes. Melanin biosynthesis begins with the hydroxylation of the amino acid tyrosine to l-DOPA, which is then oxidized to DOPAquinone. The latter moiety is polymerized to form melanin. Tyrosinase (polyphenol oxidase) plays a central role in this process as a catalyzer. Overactivity of this enzyme leads to hyperpigmentation, such as age spots, melasma, lentigines, etc., while underactivity may cause hypopigmentation of the hair. Tyrosinase activity was found to be related with Parkinson’s disease [40]. Due to the critical role of tyrosinase in melanogenesis, tyrosinase inhibitors have gained popularity in the cosmetic and pharmaceutical industries to prevent hyperpigmentation and skin-related disorders by inhibiting melanin production and controlling food browning processes [41]. Because of the side effects of the long-term or excessive use of kojic acid, a tyrosinase inhibitor, natural products have received extensive attention from researchers.

In the present study, the *in vitro* tyrosinase inhibitory effects of methanolic extracts and their fractions of *L. effusum* and *L. sinuatum* were determined by using l-DOPA as a substrate (Table 5). *n*-Hexane (IC_50_ = 148.27 ± 3.33 µg/mL) and ethyl acetate (IC_50_ = 245.56 ± 3.6 µg/mL) fractions of *L. effusum* and the ethyl acetate (IC_50_ = 295.18 ± 10.57 µg/mL) fraction of *L. sinuatum* showed noticeable antityrosinase activity. In the literature, polar extracts of some *Limonium* species were determined for their potential anti-tyrosinase activity *in vitro*. A previous study reported by Lee et al. [13] showed that myricetin 3-galactoside and hyperoside-rich fractions of *L. tetragonum* showed anti-melanogenic effects via inhibition of tyrosinase and tyrosinase-related proteins. In another study, one hundred plant extracts were screened against tyrosinase and elastase inhibitions, and among them, the 50% methanol extract of the aerial parts of *L. morisianum,* which is rich in myricetin and its glycosides, was found to be a potent tyrosinase inhibitor (56% tyrosinase inhibition tested at 50 µg/mL) [42]. Another study on the methanolic and aqueous extracts of *L. delicatulum* and *L. quesadense* leaves [27] indicated that the methanolic extracts of both species (155.87 ± 0.01 and 155.27 ± 0.01 kojic acid equivalent/g extract), which were rich in myricetin glycosides, were a promising candidate for tyrosinase inhibition. A recent study on six *Limonium* species (*L. iconicum, L. sinuatum, L. globuliferum, L. lilacinum, L. gmelinii,* and *L. bellidifolium*) reported that methanolic extracts of *L. globuliferum* and *L. iconicum* showed significant tyrosinase inhibitory activities (ranging from 153.23 to 155.67 mg kojic acid equivalent/g extract) [21].

On the other hand, quercetin glycosides, including hyperoside with IC_50_ value of 15.67 µg/mL, are accepted as being suitable for anti-melanogenesis purposes with no cytotoxicity [43]. Tannic acid exerted tyrosinase inhibition (IC_50_ value, 4.0 ± 0.01 mM), this effect was attributed its pyrogallol moiety that played a critical role in the enzyme inhibition process [44]. In addition, a molecular docking study suggested that the catechol moiety of quercetin might chelate copper in the active site of tyrosinase, causing the blocking of access of l-DOPA into the catalytic center. The mechanism of tyrosinase inhibition may explain the interaction between quercetin and tyrosinase substrates, such as l-LODA and l-tyrosine, which may lead to decreasing the formation of melanin and dopaquinone [45]. Thus, since the ethyl acetate fractions of both *Limonium* species investigated in this study were rich in tannic acid and hyperoside, and high levels of quercetin and myricetin were quantified in *L. effusum*, these phenolic compounds may be responsible for the antityrosinase activity in ethyl acetate fractions.

In addition to the simple phenolics and polyphenols in plants that showed weak to potent antityrosinase inhibitions, some other compounds, such as terpenoids, were reported to have antityrosinase activity [46]. In our study, a polar compound rich *n*-hexane showed slightly higher antityrosinase activity than the methanolic extract or other fractions. This activity may be attributed to terpenes or other lipophilic compounds.

### 2.7. Pancreatic Lipase Inhibitory Activity

Lipases are valuable and flexible enzymes, synthesized by higher organisms to break down mainly dietary oils and fats. Lipases that are present in the digestive system include tongue lipase, gastric lipase and pancreatic lipase. In the intestine, dietary fat is broken down by pancreatic lipase so that the intestine absorbs them, thereby qualifying it as the most vital enzyme for the digestion of dietary triacylglycerols. Pancreatic lipase performs the hydrolysis of 50–70% of total dietary fats. Thus, inhibiting pancreatic lipases will reduce the number of fats absorbed by the intestine [12,47]. Obesity, a chronic metabolic disease, is caused by excessive fat intake and accumulation. In managing and controlling obesity, inhibiting fat accumulation by using pancreatic lipase inhibitors is an excellent strategy and is becoming more popular. Thus, preventing obesity may decrease the incidence of obesity-related diseases and side effects. Natural products and phytomolecules have shown their potent pancreatic lipase inhibitions as anti-obesity agents [48].

The present study presented the *in vitro* pancreatic lipase inhibitions of two *Limonium* species, such as the *L. effusum* and *L. sinuatum* extracts and their fractions (Table 5). Among the tested samples, *L. sinuatum* methanol extract (IC_50_ = 162.2 ± 7.29 µg/mL) and its ethyl acetate fraction (IC_50_ = 83.76 ± 4.19 µg/mL) showed pancreatic lipase inhibition while all tested samples of *L. effusum* showed no inhibitory activity. The IC_50_ value of orlistat, a pancreatic lipase inhibitor, was calculated as 4.23 ± 0.2 µg/mL.

Data on pancreatic lipase inhibition of *Limonium* species are limited. To our knowledge, this is the first study to investigate the pancreatic lipase inhibition of *L. effusum* and *L. sinuatum.* Foddai et al. [27] reported that the aqueous extracts of *L. contortirameum* (IC_50_ = 920.4 ± 105.2 µg/mL) and *L. virgatum* (IC_50_ = 593.1 ± 56.8 µg/mL) and gallic acid, main compound, (IC_50_ = 8.4 ± 0.9 µg/mL) inhibited pancreatic lipase in a dose-dependent manner. The aqueous extracts were characterized with several phenolic compounds in their aglycon and glycoside forms, including flavones, flavanols catechins and epigallocatechin by LC-MS/MS analysis. A recent study concluded that the methanolic extract of *L. quesadense* (IC_50_ = 65 ± 7 mg orlistat equivalent/g extract) was a promising candidate as a pancreatic lipase inhibitor. Gallo(epi)catechin-*O*-gallate and its dimer, as well as myricetin-*O*-hexoside, were the most abundant compounds in *L. quesadense* [26].

Common natural sources of lipase inhibitors contain a wide range of secondary metabolites, such as flavonoids, terpenoids, saponins, alkaloids, etc. However, the mechanism of action of most isolated compounds is not known [49]. In this study, the LC-MS/MS analysis results showed that the ethyl acetate fraction of methanolic extract of *L. sinuatum* was characterized with a high content of tannic acid and hyperoside. Surprisingly, the *L. effusum* extract and its fractions showed no inhibitory activity, although it was also rich in tannic acid and hyperoside with different levels. These findings conclude that not only major compounds, such as tannic acid and hyperoside, but also minor phenolics and other types of compounds in *L. sinuatum* or their synergistic potential may contribute to pancreatic lipase inhibition. On the contrary, phenolic compounds may interact with each other; thus, the combination of phenolics may cause a decrease in the pancreatic lipase inhibitory activity, as it happens in anticholinesterase activity studies [38,39]. Further investigation is needed to understand the interaction between different phenolic compounds on the pancreatic lipase inhibitory activity.

## 3. Materials and Methods

### 3.1. Plant Materials

*L. effusum* (Boiss.) O. Kuntze was collected during the flowering period from natural population by Prof. Dr. F. Pinar Turkmenoglu and was identified by Prof. Dr. F. Pinar Turkmenoglu and Assist. Prof. Dr. Bilgehan Bilgili. The voucher specimens (No: ESSE 158002) were deposited in the Herbarium of Anadolu University Faculty of Pharmacy (ESSE). The collection cite is given as follows: *Limonium effusum* (Boiss.) O. Kuntze and: C2 Denizli: Çardak, near Acı Göl, salty sands, 830 m, F. Pınar Turkmenoglu, 20.09.2014. *L. sinuatum* (L.) Miller obtained from local bazar in Muğla, Bodrum district and deposited with the number 14003.

### 3.2. Preparation of Extracts and Fractions

The aerial parts of the plants were dried in the dark for one week at room temperature and then ground in a laboratory grinder. The powdered material was extracted with methanol at 40 °C for 3 h under stirring. The extracts were filtered and concentrated under reduced pressure, using a rotary evaporator (HeidolphLaborota 4003, Schwabach, Germany) until dried. The dried extracts were dissolved in water and partitioned with *n*-hexane, dichloromethane and ethyl acetate. Each fraction and the methanol extracts were evaporated under vacuum and stored at 4 °C [50].

### 3.3. Determination of Total Phenolic Content

The total phenolic content (TPC) was measured by the Folin–Ciocalteu colorimetric method [20], using gallic acid (Sigma-Aldrich, St. Louis, MO, USA), as a standard phenolic compound gives a crude estimation of the total phenolic compounds by measuring the amount of the substance being tested needed to inhibit the oxidation of the reagent. Briefly, 0.5 mL of the extracts (0.5 mg/mL), 2.5 mL of the Folin–Ciocalteu reagent solution (10% *v*/*v* in water) and 7.5 mL of saturated sodium carbonate (Merck, Darmstadt, Germany) (20% *w*/*v*, water) were added into a test tube. The absorbance of the resulting blue-colored solution was measured at 750 nm after incubation at 30 °C for 2 h with intermittent shaking. The total phenolic content was expressed as gallic acid equivalents (GAE) in milligram per grams of dry material.

### 3.4. Identification and Quantification of Phenolic Compounds by LC-MS/MS

In order to identify and quantify phenolic compounds, a LC-MS/MS method, which was previously reported by our research group, was used [25]. Applicability of the analytical method and the qualitative and quantitative determination of the standard compounds have already been verified. Rectilinear regression quotations and the linearity ranges of the studied standard compounds are given in Table 2. The correlation coefficients were found to be higher than 0.99. The limit of detection (LOD) and the limit of quantitation (LOQ) of the reported analytical method are shown in Table 2. For the studied compounds, the LOD ranged from 0.05 to 25.8 µg/L and LOQ ranged from 0.17 to 85.9 µg/L. Moreover, the recoveries of the phenolic compounds ranged from 96.9 to 106.2%.

### 3.5. Antioxidant Activity

#### 3.5.1. DPPH Radical Scavenging Assay

The free radical scavenging activity of the fractions was measured *in vitro* by a 2,2′-diphenyl-1-picrylhydrazyl (DPPH) assay, according to the method described earlier [28]. The stock solution was prepared by dissolving 24 mg DPPH (Sigma-Aldrich, St. Louis, MO, U.S.A.) with 100 mL methanol and stored at 20 °C until required. The working solution was obtained by diluting the DPPH solution with methanol to attain an absorbance of about 0.98 ± 0.02 at 517 nm, using the spectrophotometer. A 3 mL aliquot of this solution was mixed with 100 μL of the sample at various concentrations (1–500 μg/mL). The reaction mixture was shaken well and incubated in the dark for 30 min at room temperature. Then, the absorbance was taken at 517 nm. The scavenging activity was estimated based on the percentage of DPPH radical scavenged as the following equation:

DPPH radical scavenging activity (%) = [(A0 − A1)/A0] × 100

where A0 is the absorbance of the control at 30 min (517 nm) and A1 is the absorbance of the sample at 30 min (517 nm). BHT (Sigma-Aldrich) was used as a positive control.

#### 3.5.2. Total Antioxidant Capacity Assay

The assay was carried out using a commercial TAC assay kit (OxiSelect™ Total Antioxidant Capacity (TAC) Assay Kit, Cell Biolabs, Inc., San Diego, CA, U.S.A.). Upon reduction, the copper (I) ion further reacted with a coupling chromogenic reagent that produced a color with a maximum absorbance at 490 nm. The net absorbance values of the antioxidants were compared with a known uric acid standard curve. The absorbance values were proportional to the sample’s total reductive capacity. The results are expressed as μM copper reducing equivalents or mM uric acid equivalents. A fresh uric acid standard was prepared by weighing out the uric acid powder for a 10 mg/mL solution in 1 N NaOH. This 10 mg/mL is equivalent to a concentration of 60 mM. The 60 mM uric acid solution was used to prepare a 2 mM solution of uric acid (e.g., add 100 μL of the 60 mM uric acid standard to 2.9 mL of deionized water). Each sample was prepared using the stock solution of 10 mg/mL concentration. An initial reading was taken at 490 nm. Then, 50 μL of the 1× copper ion reagent was added and incubated for 5 min on an orbital shaker. Then, 50 μL of the stop solution was added to terminate the reaction and the plate was read again at 490 nm. All determinations were performed in triplicate and the results were averaged [24,30].

### 3.6. Antimicrobial Screening

#### 3.6.1. Test Organisms

The plant extracts and fractions were screened for their antibacterial activity against *Staphylococcus aureus* ATCC 29213, methicillin resistant *S. aureus* (MRSA) ATCC 43300, *Enterococcus faecalis* ATCC 29212, *Escherichia coli* ATCC 25922, *Pseudomonas aeruginosa* ATCC 15442 and *S. epidermidis* ATCC 35984 bacterial strains and for their antifungal activity against three *Candida* species: *C. albicans* ATCC 90028, *C. krusei* ATCC 6258 and *C. parapsilosis* ATCC 90018.

#### 3.6.2. Antimicrobial Activity Test

The broth microdilution method recommended by the Clinical and Laboratory Standards Institute (CLSI) was used to determine the antimicrobial activity [50]. The antibacterial activity test was performed in a Mueller–Hinton broth (MHB, Difco Laboratories, Detroit, MI, USA); for the antifungal test, a RPMI-1640 medium with l-glutamine (ICN-Flow, Aurora, OH, USA), buffered with MOPS buffer (ICN-Flow, Aurora, OH, USA) was used. The inoculum densities were approximately 5 × 10^5^ cfu/mL and 0.5–2.5 × 10^3^ cfu/mL for bacteria and fungi, respectively. Each plant extract was dissolved in dimethylsulfoxide. The final two-fold concentrations were prepared in the wells of the microtiter plates, between 1024 and 1 μg/mL. The microtiter plates were incubated at 35 °C for 18–24 h for bacteria and 48 h for fungi. After the incubation period, minimum inhibitory concentration (MIC) values were defined as the lowest concentration of the compounds that inhibited the visible growth of the microorganisms. Gentamicin and fluconazole were used as reference compounds for bacteria and fungi, respectively (64–0.0625 mg/mL). 

### 3.7. Anticholinesterase Inhibition Assays

Electric eel acetylcholinesterase (EC 3.1.1.7, type-VI-S), horse butyrylcholinesterase (EC 3.1.1.8), acetylthiocholine iodide, butyrylthiocholine chloride, and 5,5′-dithio-bis-nitrobenzoic acid (DTNB) were purchased from Sigma-Aldrich (St. Louis, MO, USA). Buffers and other chemicals were of extra pure analytical grade. Stock solutions of plant extracts (10 mg/mL) were prepared in DMSO. Inhibition was determined spectrophotometrically by modifying the method of Ellman [51]. In this method, 150 µL of 0.1 M potassium phosphate buffer (pH 8.0), 20 µL enzyme preparation and 10 µL sample at different concentrations dissolved in DMSO were mixed and incubated for 30 min. A total of 10 µL of DTNB (0.5 mM) was added and the reaction was then started by adding 10 µL of acetylthiocholine iodide (0.71 mM), or butyrylthiocholine chloride (0.2 mM) was used as a substrate, while all the other reagents and conditions were the same. The hydrolysis of acetylthiocholine iodide or butyrylthiocholine chloride was determined by monitoring the formation of the yellow 5-thio-2-nitrobenzoate anion as a result of the reaction with DTNB with thiocholines, catalyzed by enzymes at a wavelength of 412 nm, using a Shimadzu 1601 PC spectrophotometer equipped with a Peltier unit (extinction coefficients of 14.2 mM^−1^cm^−1^ and 13.6 mM^−1^cm^−1^ were used in order to calculate the acetylcholinesterase and butyrylcholinesterase enzyme activities, respectively). DMSO was used as a negative control. Donepezil was used as a positive control. Inhibition percentage was calculated according to the Michaelis–Menten model.

### 3.8. Tyrosinase Inhibition Assay

The method reported by Likhitwitayawuid et al. [52] was employed with slight modifications. The samples were prepared with 100 mM phosphate buffer (6.8 pH) (PB). For each concentration of the sample solution, four wells were designated as A, B, C and D, and each contained a reaction mixture (40 µL) as follows: A, 20 µL of PB + 20 µL of tyrosinase (200 U/mL) in PB (TYR); B, 40 µL of PB; C, 20 µL of TYR + 20 µL of the sample; D, 20 µL of the sample + 20 µL of PB. The contents of each well were mixed in a 96-well microtiter plate and incubated at 37 °C for 10 min. Then, 5 mM of l-DOPA (160 µL) was added. After second incubation at 37 °C for 10 min, the absorbances were measured at 475 nm. The percentage inhibition of the tyrosinase activity was calculated by the following equation:

[(A − B) − (C − D)/(A − B)] × 100.

### 3.9. Pancreatic Lipase Inhibition Assay

The pancreatic lipase assay was employed according to the method described by McDougall et al. [53] with slight modifications. Porcine pancreas lipase Type II (PPL) (Sigma product L3126) was dissolved in assay buffer (100 mM Tris-HCl, pH 8.2) at 10 mg/mL. Then, the supernatant was used after centrifugation at 5000 rpm for 10 min. *p*-nitrophenyl laurate (*p*-NPL) was used as the substrate and 0.08% *w*/*v p*-NPL stock solution was prepared in 5 mM sodium acetate buffer (pH 5.0) (NaOAc) containing 1% Triton X-100. The extracts were dissolved in ultra-pure water. A total of 10 µL sample + 80 µL NaOAc + 30 µL of PPL (A, sample), 10 µL sample + 110 µL NaOAc (B, sample blank), 30 µL PPL + 90 µL NaOAc (C, enzyme control), and 120 µL NaOAc (D, solvent blank) were mixed in 96-well microtiter plate and incubated at 37 °C for 10 min. Then, 80 µL of the substrate solution was added and incubated at 37 °C for 10 min. Then, 80 µL of *p*-NPL was added and incubated at 37 °C for 2 h. After incubation, the absorbance was measured at 400 nm and the percentage inhibition of the pancreatic lipase activity was calculated by the following equation:

[(C − D) − (A − B)/(C − D)] × 100.

### 3.10. Statistical Analysis

All the experiments were carried out in triplicate, and data are expressed as means ± standard deviation (SD) for triplicate determinations (*n* = 3). Statistical analyses were performed using Sigmaplot 14.0 software (Systat Software, Inc., San Jose, CA, U.S.A.). The differences between the samples were analyzed by one-way analysis of variance (ANOVA) and Duncan’s multiple-range tests with *p* < 0.05 being considered significant. IC_50_ values were calculated by regression analysis.

## 4. Conclusions

The results of the present study exhibited important data regarding phenolic composition, and the antioxidant, antimicrobial, anticholinesterase, antiyrosinase and pancreatic lipase inhibitory potential of two edible *Limonium* species, *L. effusum* and *L. sinuatum*. Phytochemical analysis indicated high amounts of tannic acid and hyperoside, which were detected in the *Limonium* species as major compounds for the first time, regarding that both *L. effusum* and *L. sinuatum* are new sources for these natural compounds. Both species are rich in total phenolics and potent antioxidant activities higher than BHT were detected in different assays. The antimicrobial activity assay indicated that both species are more active with Gram-positive bacteria than Gram negatives. Significant growths inhibition against fungi was also determined. In terms of anticholinesterase activity, although both species inhibited AChE and BChE, selective AChE inhibitions took attention. *L. sinuatum* exhibited more vigorous AChE inhibitory activity than *L. effusum*. While both species showed antityrosinase activity, only *L. sinuatum* inhibited pancreatic lipase. Generally, the most potent activity was observed in the ethyl acetate fractions.

The overall results indicate that *L. effusum* and *L. sinuatum* are rich sources of bioactive compounds, and both species can be used in the development of new pharmaceuticals, cosmetics and nutraceuticals. However, further studies, particularly in vivo tests, are needed to understand their activities in biological systems.

## Figures and Tables

**Table 1:** Total phenolic content and antioxidant activity of *Limonium* extracts and their fractions.

Sample	Total Phenolic Content	DPPH Scavenging Assay	Total Antioxidant Capacity	
	mg GAE/g Extract ^1^	IC_50_ (µg/mL)	UAE ^2^ (mM)	CRE ^3^ (µM)
***L. effusum***				
*n*-Hexane	109.08 ± 4.26 ^c^	13.92 ± 0.06 ^a^	0.287 ± 0.007 ^f^	629.32 ± 15.44 ^f^
Dichloromethane	111.31 ± 3.17 ^c^	227.97 ± 15.83 ^e^	0.131 ± 0.006 ^b^	287.62 ± 13.81 ^b^
Ethyl acetate	522.82 ± 5.67 ^h^	30.15 ± 0.82 ^b^	0.453 ± 0.007 ^g^	990.75 ± 14.86 ^g^
Water	88.37 ± 3.12 ^b^	144.16 ± 7.26 ^c^	0.136 ± 0.001 ^bc^	296.94 ± 2.35 ^bc^
Methanol	210.91 ± 2.68^f^	28.72 ± 0.79 ^b^	0.226 ± 0.003 ^e^	495.07 ± 6.17 ^e^
***L. sinuatum***				
*n*-Hexane	93.68 ± 3.41 ^b^	180.50 ± 6.89 ^d^	0.153 ± 0.004 ^c^	335.80 ± 8.06 ^c^
Dichloromethane	124.11 ± 4.27 ^d^	174.06 ± 5.14 ^d^	0.193 ± 0.002 ^d^	421.55 ± 4.76 ^d^
Ethyl acetate	274.87 ± 1.87 ^g^	5.27 ± 0.002 ^a^	0.836 ± 0.016 ^j^	1829.47 ± 35.27 ^j^
Water	75.98 ± 0.88 ^a^	160.79 ± 8.02 ^d^	0.041 ± 0.002 ^a^	89.43 ± 3.63 ^a^
Methanol	189.62 ± 1.43 ^e^	30.79 ± 0.75 ^b^	0.176 ± 0.005 ^d^	384.65 ± 11.47 ^d^
BHT *	-	32.63 ± 0.63 ^b^	0.540 ± 0.022 ^h^	1182.99 ± 48.37 ^h^

The values are given as means of triplicate analyses, results are represented as means ± standard deviation (*n* = 3). Means in the same column followed by the same letter are not significantly different at *p* < 0.05. * Positive control. ^1^ Gallic acid equivalent. ^2^ Uric acid equivalent. ^3^ Copper reducing equivalent.

**Table 2:** LC-MS/MS analysis of phenolic compounds detected in *L. effusum* and *L. sinuatum*.

No	Analytes	RT ^a^	Parent Ion (*m*/*z*) ^b^	Ionization Mode	R^2 c^	RSD% ^d^	Linearity Range (mg/L)	LOD/LOQ (µg/L) ^e^	Recovery (%)	U ^f^	Quantification (µg/g Extract) ^g^	
											*L. effusum*	*L. sinuatum*
1	Quinic acid	3.32	190.95	Neg	0.9927	0.0388	250–10,000	22.3/74.5	103.3	4.8	636.08 ± 30.5	D.^h^
2	Malic acid	3.54	133.05	Neg	0.9975	0.1214	250–10,000	19.2/64.1	101.4	5.3	1007.66 ± 53.3	410.69 ± 21.7
3	*trans*-Aconitic acid	4.13	172.85	Neg	0.9933	0.3908	250–10,000	15.6/51.9	102.8	4.9	D.	N.D. ^h^
4	Gallic acid	4.29	169.05	Neg	0.9901	0.4734	25–1000	4.8/15.9	102.3	5.1	5798.56 ± 295.6	4237.76 ± 216.1
5	Chlorogenic acid	5.43	353	Neg	0.9932	0.1882	250–10,000	7.3/24.3	99.7	4.9	D.	N.D.
6	Protocatechuic acid	5.63	152.95	Neg	0.9991	0.5958	100–4000	25.8/85.9	100.2	5.1	685.93 ± 34.9	185.14 ± 9.4
7	Tannic acid	6.46	182.95	Neg	0.9955	0.9075	100–4000	10.2/34.2	97.8	5.1	71,439.56 ± 3643.3	105,453.5 ± 5328.1
8	*trans*-caffeic acid	7.37	178.95	Neg	0.9942	1.0080	25–1000	4.4/14.7	98.6	5.2	237.24 ± 12.3	476.49 ± 24.75
9	Vanillin	8.77	151.05	Neg	0.9995	0.4094	250–10,000	10.1/33.7	99.2	4.9	N.D.	N.D.
10	*p*-Coumaric acid	9.53	162.95	Neg	0.9909	1.1358	100–4000	15.2/50.8	98.4	5.1	743.06 ± 37.9	236.02 ± 12.1
11	Rosmarinic acid	9.57	358.9	Neg	0.9992	0.5220	250–10,000	10.4/34.8	101.7	4.9	N.D.	N.D.
12	Rutin	10.18	609.1	Neg	0.9971	0.8146	250–10,000	17.0/56.6	102.2	5.0	N.D.	60.74 ± 3.1
13	Hesperidin	9.69	611.1	Poz	0.9973	0.1363	250–10,000	21.6/71.9	100.2	4.9	N.D.	N.D.
14	Hyperoside	10.43	463.1	Neg	0.9949	0.2135	100–4000	12.4/41.4	98.5	4.9	14,006.90 ± 686.1	1708.51 ± 83.6
15	4-OH Benzoic acid	11.72	136.95	Neg	0.9925	1.4013	25–1000	3.0/10.0	106.2	5.2	126.60 ± 6.5	124.04 ± 6.4
16	Salicylic acid	11.72	136.95	Neg	0.9904	0.6619	25–1000	4/13.3	106.2	5.0	119.34 ± 5.9	121.59 ± 6.0
17	Myricetin	11.94	317	Neg	0.9991	2.8247	100–4000	9.9/32.9	106.0	5.9	1646.93 ± 97.1	N.D.
18	Fisetin	12.61	284.95	Neg	0.9988	2.4262	100–4000	10.7/35.6	96.9	5.5	N.D.	N.D.
19	Coumarin	12.52	146.95	Poz	0.9924	0.4203	100–4000	9.1/30.4	104.4	4.9	N.D.	N.D.
20	Quercetin	14.48	300.9	Neg	0.9995	4.3149	25–1000	2.0/6.8	98.9	7.1	975.24 ± 69.2	94.23 ± 6.7
21	Naringenin	14.66	270.95	Neg	0.9956	2.0200	25–1000	2.6/8.8	97.0	5.5	9.30 ± 0.5	15.61 ± 0.8
22	Hesperetin	15.29	300.95	Neg	0.9961	1.0164	25–1000	3.3/ 11.0	102.4	5.3	N.D.	N.D.
23	Luteolin	15.43	284.95	Neg	0.9992	3.9487	25–1000	5.8/19.4	105.4	6.9	67.16 ± 4.6	61.55 ± 4.2
24	Kaempferol	15.43	284.95	Neg	0.9917	0.5885	25–1000	2.0/6.6	99.1	5.2	68.17 ± 3.5	59.97 ± 3.1
25	Apigenin	17.31	268.95	Neg	0.9954	0.6782	25–1000	0.1/0.3	98.9	5.3	751.20 ± 39.8	7.49 ± 0.4
26	Rhamnetin	18.94	314.95	Neg	0.9994	2.5678	25–1000	0.2/0.7	100.8	6.1	N.D.	N.D.
27	Chrysin	21.18	253	Neg	0.9965	1.5530	25–1000	0.05/0.17	102.2	5.3	N.D.	N.D.

^a^ Retention time, ^b^ Parent ion (*m*/*z*)*:* Molecular ions of the standard compounds (mass to charge ratio), ^c^ R^2^: coefficient of determination, ^d^ RSD: relative standard deviation, ^e^ LOD/LOQ (µg/L): Limit of detection/Limit of quantification, ^f^ U (%): Percent relative uncertainty at 95% confidence level (k = 2), ^g^ Values in µg/g (*w/w*) of ethyl acetate fractions, ^h^N.D: not detected, D: peak observed, concentration is lower than the LOQ but higher than the LOD.

**Table 3:** Minimum inhibitory concentration (MIC) values (μg/mL) of *L. effusum* and *L. sinuatum* and reference antibiotics, gentamicin and fluconazole *.

Sample	MIC (µg/mL)								
	Bacteria						Fungi		
	*S. aureus* ATCC 29213	MRSA ATCC 43300	*S. epidermidis* ATCC 35984	*E. faecalis* ATCC 29212	*E. coli* ATCC 25922	*P. aeruginosa* ATCC 15442	*C. albicans* ATCC 90028	*C. krusei* ATCC 6258	*C. parapsilosis* ATCC 90018
***L. effusum***									
*n*-Hexane	512	512	128	64	256	256	128	128	64
Dichloromethane	1024	1024	256	512	512	256	256	128	64
Ethyl acetate	512	512	128	128	512	256	256	128	64
Water	256	512	256	256	512	256	128	128	64
Methanol	256	512	256	256	512	256	128	128	128
***L. sinuatum***									
*n*-Hexane	16	32	16	16	512	256	128	64	64
Dichloromethane	512	1024	256	512	512	256	128	128	64
Ethyl acetate	64	128	64	32	512	256	64	64	32
Water	1024	512	256	256	512	256	256	128	64
Methanol	256	512	256	64	512	256	128	64	64
Gentamicin	<1	-	-	4	<1	0.5	-	-	-
Fluconazole	-	-	-	-	-	-	1	32	4

* Results are the mean of three experiments.

**Table 4:** Acetylcholinesterase and butyrylcholinesterase activity of *L. effusum* and *L. sinuatum*.

Sample	IC_50_ (µg/mL)		Selectivity Index (AChE/BChE)
	AChE	BChE	
***L. effusum***			
*n*-Hexane	7.353 ± 1.15 ^b^	224.03 ± 25.78 ^a^	0.032
Dichloromethane	6.081 ± 0.87 ^b^	1943.00 ± 418.61 ^ab^	0.003
Ethyl acetate	35.594 ± 2.13 ^c^	1521.00 ± 359.21 ^ab^	0.023
Water	38.697 ± 3.56 ^c^	-	-
Methanol	2.808 ± 0.58 ^ab^	2741.50 ± 478.71 ^ab^	0.001
***L. sinuatum***			
*n*-Hexane	5.987 ± 0.65 ^b^	308.72 ± 9.65 ^a^	0.019
Dichloromethane	39.209 ± 3.26 ^c^	>1000 ^c^	>0.040
Ethyl acetate	5.634 ± 0.93 ^b^	4022.00 ± 889.54 ^b^	0.001
Water	0.199 ± 0.009 ^a^	14,882.50 ± 2689.13 ^d^	1.33 × 10^−5^
Methanol	6.544 ± 1.09 ^b^	14,666.00 ± 2029.39 ^d^	4.46 × 10^−4^
Donepezil *	0.0035 ± 0.0007 ^a^	0.0027 ± 0.0005 ^a^	1.27

Data are given as mean ± SD (*n* = 3*)*. Means in the same column followed by the same letter are not significantly different at *p* < 0.05. * Positive control. (-): not detected. Selectivity index (SI) was calculated as SI (IC_50_AChE/IC_50_BChE). Selectivity toward AChE increases as the corresponding SI decreases.

**Table 5:** Tyrosinase and pancreatic lipase inhibition of extracts.

Sample	IC_50_ (µg/mL)	
	Tyrosinase	Pancreatic Lipase
***L. effusum***		
*n*-Hexane	148.27 ± 3.33 ^b^	-
Dichloromethane	-	-
Ethyl acetate	245.56 ± 3.6 ^d^	-
Water	-	-
Methanol	-	-
***L. sinuatum***		
*n*-Hexane	-	-
Dichloromethane	-	-
Ethyl acetate	295.18 ± 10.57 ^e^	83.76 ± 4.19 ^b^
Water	-	-
Methanol	-	162.2 ± 7.29 ^c^
Kojic acid *	14.28 ± 0.6 ^a^	-
Orlistat *	-	4.23 ± 0.2 ^a^

Data are expressed as mean ± SD (*n* = 3*)*. Means in the same column followed by the same letter are not significantly different at *p* < 0.05. * Positive control. (-): not detected.

## Data Availability

The data presented in this study are available within the article.

## Sample Availability

Samples of the plants, extracts and fractions are available from the authors.
